# Effects of Immune Activation during Early or Late Gestation on *N*-Methyl-d-Aspartate Receptor Measures in Adult Rat Offspring

**DOI:** 10.3389/fpsyt.2017.00077

**Published:** 2017-09-04

**Authors:** Tasnim Rahman, Katerina Zavitsanou, Tertia Purves-Tyson, Lauren R. Harms, Crystal Meehan, Ulrich Schall, Juanita Todd, Deborah M. Hodgson, Patricia T. Michie, Cyndi Shannon Weickert

**Affiliations:** ^1^Faculty of Medicine, School of Psychiatry, University of New South Wales, Sydney, NSW, Australia; ^2^Neuroscience Research Australia, Randwick, NSW, Australia; ^3^Schizophrenia Research Institute, at Neuroscience Research Australia, Randwick, NSW, Australia; ^4^School of Psychology, University of Newcastle, Callaghan, NSW, Australia; ^5^Priority Centre for Brain and Mental Health Research, University of Newcastle, Newcastle, NSW, Australia; ^6^Hunter Medical Research Institute, Newcastle, NSW, Australia; ^7^School of Medicine and Public Health, University of Newcastle, Callaghan, NSW, Australia

**Keywords:** striatum, NMDA, polyriboinosinic:polyribocytidylic acid, maternal immune activation, Wistar rat, schizophrenia, rat model

## Abstract

**Background:**

Glutamatergic receptor [*N*-methyl-d-aspartate receptor (NMDAR)] alterations within cortex, hippocampus, and striatum are linked to schizophrenia pathology. Maternal immune activation (MIA) is an environmental risk factor for the development of schizophrenia in offspring. In rodents, gestational timing of MIA may result in distinct behavioral outcomes in adulthood, but how timing of MIA may impact the nature and extent of NMDAR-related changes in brain is not known. We hypothesize that NMDAR-related molecular changes in rat cortex, striatum, and hippocampus are induced by MIA and are dependent on the timing of gestational inflammation and sex of the offspring.

**Methods:**

Wistar dams were treated the with viral mimic, polyriboinosinic:polyribocytidylic acid (polyI:C), or vehicle on either gestational day 10 or 19. Fresh-frozen coronal brain sections were collected from offspring between postnatal day 63–91. Autoradiographic binding was used to infer levels of the NMDAR channel, and NR2A and NR2B subunits in cortex [cingulate (Cg), motor, auditory], hippocampus (dentate gyrus, cornu ammonis area 3, cornu ammonis area 1), and striatum [dorsal striatum, nucleus accumbens core, and nucleus accumbens shell (AS)]. NR1 and NR2A mRNA levels were measured by *in situ* hybridization in cortex, hippocampus, and striatum in male offspring only.

**Results:**

In the total sample, NMDAR channel binding was elevated in the Cg of polyI:C offspring. NR2A binding was elevated, while NR2B binding was unchanged, in all brain regions of polyI:C offspring overall. Male, but not female, polyI:C offspring exhibited increased NMDAR channel and NR2A binding in the striatum overall, and increased NR2A binding in the cortex overall. Male polyI:C offspring exhibited increased NR1 mRNA in the AS, and increased NR2A mRNA in cortex and subregions of the hippocampus.

**Conclusion:**

MIA may alter glutamatergic signaling in cortical and hippocampal regions *via* alterations in NMDAR indices; however, this was independent of gestational timing. Male MIA offspring have exaggerated changes in NMDAR compared to females in both the cortex and striatum. The MIA-induced increase in NR2A may decrease brain plasticity and contribute to the exacerbated behavioral changes reported in males and indicate that the brains of male offspring are more susceptible to long-lasting changes in glutamate neurotransmission induced by developmental inflammation.

## Introduction

Epidemiological investigations implicate gestational inflammation as a significant risk factor in the manifestation of psychiatric disorders, including schizophrenia, in offspring (1). Studies suggest either the first (2) or second (1) trimester as the critical window of vulnerability for maternal infection to increase schizophrenia risk in people. Maternal immune activation (MIA), as modeled in rodents and non-human primates, suggests pro-inflammatory cytokines that result from MIA, rather than the pathogen itself, are critical in the development of schizophrenia-like behaviors and neuropathology in adult offspring (3–6). Deficits in sensorimotor gating and working memory are prevalent in schizophrenia (7, 8) and found in MIA rodents. The use of MIA in animal models has therefore been pivotal to establish a causal link between gestational inflammation and the development of neuropsychiatric-related phenotypes; however, only limited changes in brain cytokines are still found in the adult (9), suggesting that molecular alterations in neurotransmitter pathways including monoamines (10, 11) and GABA (12) may be more long lasting. Since pharmacological manipulations of glutamate neurotransmission are known to cause deficits in sensorimotor gating and in working memory, which are reminiscent of changes found in schizophrenia, we predict that widespread changes in glutamate receptors may be found in adult offspring of mothers who experienced immune activation while pregnant. While there is a report of a reduction in one subunit of the *N*-methyl-d-aspartate receptor (NMDAR) in one brain region (the hippocampus) (13), there are still gaps in our knowledge regarding the putative changes in other NMDAR subunits and the extent of NMDAR changes outside the hippocampus. It is important to determine the nature and extent of NMDAR changes more fully to not only correlate them with behavior but to also aid in the design of treatments aimed to either prevent or ameliorate changes in NMDAR-mediated neurotransmission that may underlie symptoms and cognitive deficits resulting from developmental overactivation of the immune system. This knowledge will expand our understanding of how gestational inflammation impacts glutamatergic signaling in psychiatric disorders with a neurodevelopmental etiology.

The timing of MIA can elicit specific behavioral phenotypes in rodent offspring during development and adulthood (6, 14). This phenomenon has been demonstrated in a mouse model that used the viral mimic, synthetic double-stranded RNA polyriboinosinic:polyribocytidilic acid (polyI:C, henceforth polyI:C offspring). Early polyI:C exposure on gestational day (GD) 9 results in adult offspring with behaviors that are used to index schizophrenia-like behaviors, such as sensorimotor gating deficits (13) and primarily dopaminergic-related behavioral alterations concomitant with a reduction in dopamine receptor D1 levels in cortex (13, 15, 16). Late polyI:C exposure on GD17 results in offspring with behavioral changes consistent with schizophrenia-like deficits in cognitive function, including working memory and reversal learning deficits (13, 17). Late polyI:C exposure results in offspring with changes in the hippocampus that include NMDAR-related alterations (13). In rats, mid-late gestation (GD14–17) MIA produces a range of schizophrenia-like phenotypes that include both increased and decreased locomotor response to the NMDAR antagonist MK-801 (18–21), deficits in sensorimotor gating (22–25), impaired cognition (23, 26), and reduced brain volume in cortical, hippocampal, and striatal regions (27, 28). We have recently reported that early (GD10) and late (GD19) MIA in rats, at developmentally equivalent times to mice (29), may not result in differential schizophrenia-like behaviors. We found that males with early polyI:C exhibit sensorimotor gating deficits and that males with late (GD19) polyI:C exhibit sensorimotor gating and working memory deficits (30), suggesting that there may be long-lasting alterations in cortical glutamate neurotransmission especially after late MIA.

A key glutamate receptor involved in learning and memory is the NMDAR. NMDAR hypofunction is a prevailing hypothesis in the pathophysiology of schizophrenia (31) and NMDAR1 is reduced in the frontal cortex of people with schizophrenia compared to controls (32). NMDARs are ionotropic glutamate receptors essential for synaptic plasticity throughout the brain (33). NMDARs consist of two obligatory NR1 subunits and two regulatory subunits (i.e., NR2A and/or NR2B) functioning as a membrane heterotetramer (34). NR2B-rich NMDARs predominate throughout the brain during early development, and NR2A-containing NMDARs gradually increase expression after birth (34). NMDARs rich in NR2A have shorter channel opening time and an increased probability to open compared to NR2B-rich NMDARs (35, 36). Additionally, the ratio between NR2A- and NR2B-containing NMDARs in the hippocampus is critical in activity-dependent plasticity, where increased NR2A:NR2B is associated with less plasticity and reduced signal transduction (37–39). PolyI:C offspring exhibit decreased neonatal neuronal excitability (40), and decreased NR1 immunoreactivity (13) and synaptic plasticity in the hippocampus in adulthood. The extent to which other NMDAR regulatory subunits (2A and 2B) are changed due to MIA and the relationship of NMDAR changes to the time of exposure and sex of the offspring will provide information needed to help design ways to ameliorate the neurobiological impact of MIA.

In the current study, we sought to elucidate the effects of early (GD10) versus late (GD19) polyI:C treatment on NMDA receptor subunits in a range of cortical, hippocampal, and striatal subregions of male and female adult offspring. We hypothesize that MIA will induce NMDAR-related molecular changes in rat cortex, striatum, and hippocampus in adult offspring and changes will be dependent both on the timing of gestational inflammation and the sex of the offspring. We specifically aimed to extend the scope of previous MIA studies, which examined NR1 mRNA only (13), to include quantitative binding assays of the whole NMDA channel, and NR2A and NR2B subunits. As our largest changes were primarily NMDAR binding alterations in male polyI:C offspring, we investigated related mRNAs in the same brain regions from male offspring only.

## Materials and Methods

### Animals and Prenatal PolyI:C Administration

Experiments were performed in accordance with the National Health and Medical Research Council’s *Australian code for the care and use of animals for scientific purposes*. The current study was approved by the University of Newcastle’s Animal Care and Ethics Committee (Approval number A-2009-108). Rats were sourced from the University of Newcastle’s Central Animal House and housed with *ad libitum* food and water and 12 h light exposure in the University of Newcastle’s Behavioral Sciences Animal Facility.

Postnatal day (PND) 70–90 Wistar rats were time-mated and day of vaginal plug detection was designated as GD0. Pregnant rats were assigned to two groups: GD10 (control, *n* = 8) or GD19 (*n* = 7). On the appropriate GD, pregnant rats were weighed, lightly anesthetized with isoflurane, and injected intravenously through the tail vein with 0.1 M phosphate-buffered saline (PBS) (control; *n* = 8) or 4 mg/mL of polyI:C (P9582, Sigma-Aldrich; *n* = 7) in PBS at a volume of 1 mL/kg body weight. To confirm successful MIA, saphenous vein blood samples were collected 2 h after treatment injections. Plasma was used for interleukin-6 (IL-6) measurement using rat IL-6 Quantikine ELISA (R&D Systems, MN, USA). PolyI:C treated dams had significantly increased IL-6 levels (624.7 ± 57.0 pg/mL) compared to saline-injected dams (68.4 ± 57.0 pg/mL) [*F*_(1,8)_ = 47.646, *p* < 0.001]. There was no effect of gestational timing [*F*_(1,8)_ = 0.218, *p* > 0.05] or interaction between gestational timing and treatment [*F*_(1,8)_ = 0.211, *p* > 0.05] on maternal plasma IL-6 levels (data not shown).

**Figure 1:**
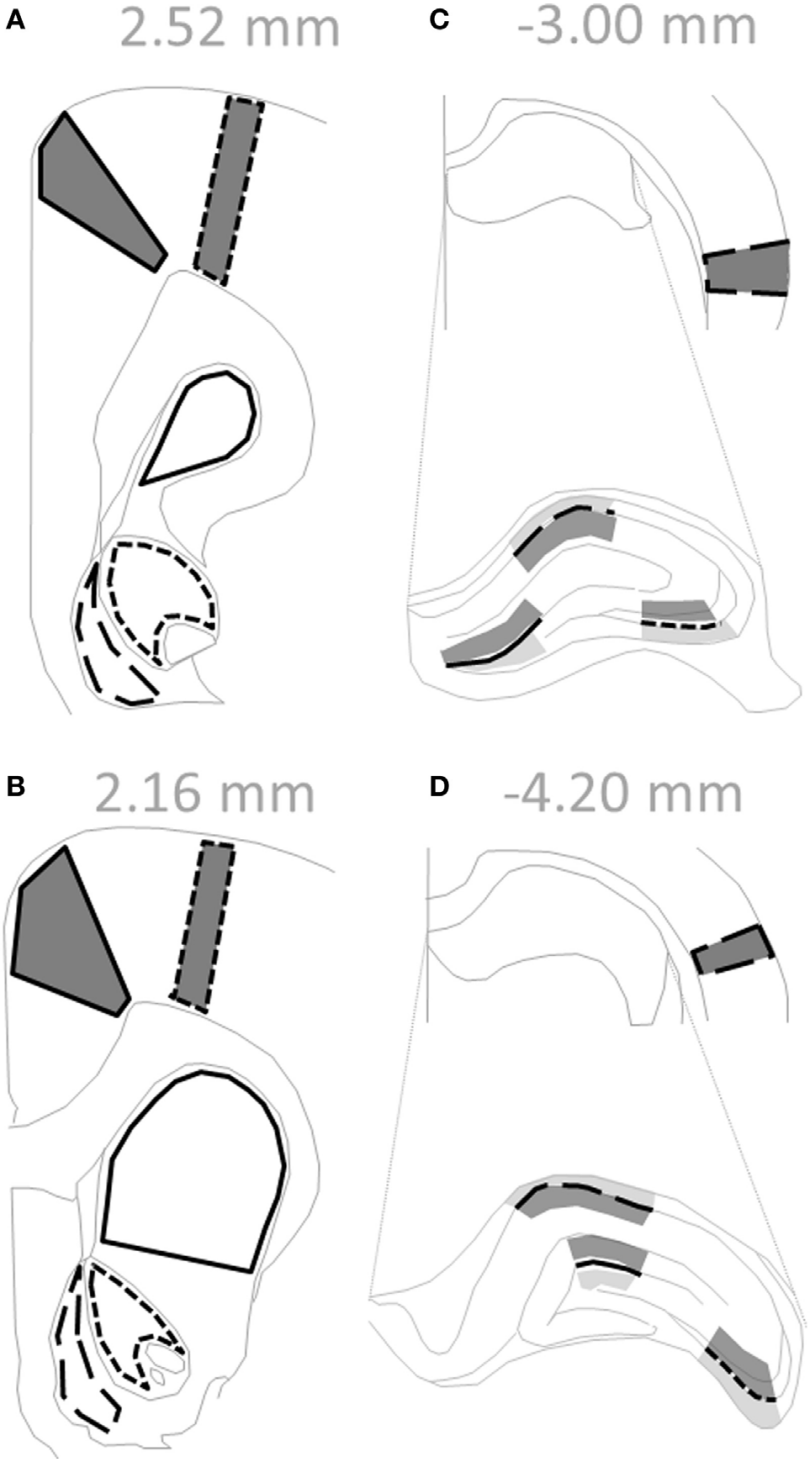
Regions within coronal rat sections used for autoradiographic binding and *in situ* hybridization. **(A–D)** Solid gray polygons outlined in black depict cortical subregions quantified: cingulate [**(A,B)** solid line], motor [**(A,B)** short dashed line], and auditory [**(C,D)** long dashed line]. **(C,D)** Hippocampus is enlarged to delineate subregions, where black lines depict: dentate gyrus (solid line), cornu ammonis field 3 (short dashed line), and cornu ammonis field 1 (long dashed line). Shaded regions indicate levels quantified for binding data: afferent (dark gray; molecular layer, stratum radiatum) and efferent (light gray; polymorph layer, stratum oriens). **(A,B)** Empty black outlines depict striatal subregions quantified: dorsal striatum (solid line), nucleus accumbens core (short dashed line), and nucleus accumbens shell (long dashed line). Text in gray indicates Bregma level of section. Adapted from Paxinos and Watson sixth edition Rat Atlas (41).

Offspring were weaned on PND 21, separated into same-sex cages in pairs, and euthanized by isoflurane anesthesia and decapitation between PND 63–91 (*n* = 6–8 for each subgroup). At time of euthanasia, there was no effect of treatment on weight between control (350 ± 6 g) and polyI:C offspring (341 ± 7 g) [*F*_(1,50)_ = 0.975, *p* > 0.05]. There was a significant effect of sex on weight where female offspring (264 ± 6 g) weighed less than males (426 ± 7 g) [*F*_(1,50)_ = 302.0, *p* < 0.05]. There were no other interaction effects [*F*_(1,50)_ < 1.50, *p* > 0.05; data not shown]. Whole brains were snap-frozen in isopentane at −40°C and stored at −80°C. Coronal sections (14 µm; Figure 1) were prepared using a cryostat (Leica, Wetzlar, Germany) and mounted onto gelatin-coated glass slides.

### *In Situ* Radioligand Binding

Receptors were identified using autoradiography and tritiated specific ligands. Following the autoradiographic assays (Table 1), slides were dipped in ice-cold distilled H_2_O, dried in a stream of cold air, and exposed to BioMax MR (Kodak, Rochester, NY, USA) autoradiographic film for 75 ([^3^H]MK-801), 76 ([^3^H]CGP39653), or 69 ([^3^H]Ifenprodil) days with tritium standards (American Radiolabeled Chemicals, St. Louis, MO, USA). Films were developed using standard procedures.

**Table 1:** Summary of autoradiographic binding methods.

Radioligand (target)	Preincubation	Specific binding conditions	Non-specific binding condition	Wash
[^3^H]MK-801 (42) (*N*-methyl-d-aspartate receptor channel)		2.5 h at RT	20 µM non-tritiated MK-801 (Sigma)	2 × 20 min at 4°C
		30 mM HEPES buffer with 100 µM glycine, 100 µM glutamate, 1 mM EDTA, and 20 nM [^3^H]MK-801 (s.a. 17.1 Ci/mmol, PerkinElmer, USA), pH 7.5		30 mM HEPES buffer with 1 mM EDTA, pH 7.5
[^3^H]CGP39653 (43) (NR2A subunit)	45 min at RT	45 min at RT	1 mM l-glutamic acid	30 s at 4°C
	50 mM Tris–HCl, pH 8	50 mM Tris–HCl with 20 nM [^3^H]CGP39653, pH 8		50 mM Tris–HCl, pH 8
[^3^H]Ifenprodil (NRB subunit)		3 h at 4°C	10 µM non-tritiated Ifenprodil	3 × 5 min at 4°C
		50 mM Tris–HCI buffer with 3 μM R(+)-3-(3-hydroxyphenyl)-*N*-propylpiperidine hydrochloride, 30 µM GBR-12909, 100 µM 20 nM [^3^H]Ifenprodil trifluoperazine, pH 7.4		50 mM Tris–HCl buffer, pH 7.4

### Quantification of Autoradiographic Images

Developed films were digitized and calibrated using the NIH imaging software (v1.56^1^) to produce nCi/mg tissue equivalent values based on the standard Rodbard curve obtained from the ^3^H standards (American Radiolabeled Chemicals, St. Louis, MO, USA). Optical density values were quantitated using ImageJ (v1.48^2^). Non-specific binding was at background levels for all ligands examined; therefore, total binding values were averaged from between two and four consecutive sections for each measure. Cortical [cingulate (Cg), motor (M1), auditory (Aud)], hippocampal [dentate gyrus (DG), cornu ammonis area 3 (CA3), cornu ammonis area 1 (CA1)], and striatal [dorsal striatum (DS), nucleus accumbens core (AC), nucleus accumbens shell (AS)] regions were quantified (Figure 1). For hippocampus, afferent and efferent (neuropil) regions were quantified for binding, while the soma layer was quantified for mRNA. All brain regions were identified as per Paxinos and Watson sixth edition Rat Atlas (41).

### *In Situ* Hybridization

Sections from control and MIA male offspring were investigated for related mRNAs. Riboprobes (Table 2) were generated with ^35^S-UTP (PerkinElmer) using an *in vitro* transcription kit (Promega, Madison, WI, USA). *In situ* hybridization was performed as previously described (44), using 5 ng/mL radiolabeled riboprobes in hybridization buffer, and ^35^S-UTP labeled sense riboprobes as a negative control. Slides were exposed to BioMax MR (Kodak, Rochester, NY, USA) autoradiographic film (details in Table 2) alongside a ^14^C standard slide (American Radiolabeled Chemicals, St. Louis, MO, USA). Quantification of mRNAs was completed as mentioned above (see Quantification of Autoradiographic Images) with the standard Rodbard curve from the ^14^C standard slide.

**Table 2:** *In situ* hybridization riboprobe details.

Target gene	Bp region from origin (0)	LOCUS ID	Autoradiographic exposure time on film (days)	Specific activity (cpm/μg) of riboprobe	
				Sense (+)	Antisense (−)
GRIN1 (NR1)	1,840–2,081	NM_008169.3	10	1.69 × 10^9^	1.42 × 10^9^
GRIN2A (NR2A)	3,218–3,418	NM_000833.2	21	1.61 × 10^9^	1.87 × 10^9^

### Statistical Analysis

Analysis was performed with IBM SPSS statistics (v23). Graphs were plotted using GraphPad Prism (v6). Data for each measure in each region (cortex, hippocampus, striatum) were analyzed using repeated measures two-way analysis of variance (RM-ANOVA) separately. Within-subject factors were the three subregions. Between-subject factors were prenatal treatment (polyI:C or vehicle), offspring sex (male or female), and gestational timing (GD10 or GD19) for the binding, and prenatal and gestational timing for the mRNAs (in male offspring only). Fisher’s least significant differences were used for pairwise comparisons by treatment when ANOVA analyses were significant. The Greenhouse Geisser correction was used if Mauchley’s sphericity test was violated for within-subjects interaction effects. Overall treatment effects or effects of sex, region, or GD of exposure are presented graphically only when a significant effect or interaction was identified. In all cases, data are expressed as the mean ± SEM and *p* ≤ 0.05 was deemed statistically significant. NR2A:NR2B ratio for hippocampus was calculated by division of [^3^H]CGP39653 binding values (NR2A subunit) with [^3^H]Ifenprodil binding values (NR2B subunit) in each subregion and level for each animal.

## Results

### Effect of Maternal PolyI:C Treatment and Timing on Ligand Binding in Adult Male and Female Offspring

**Figure 2:**
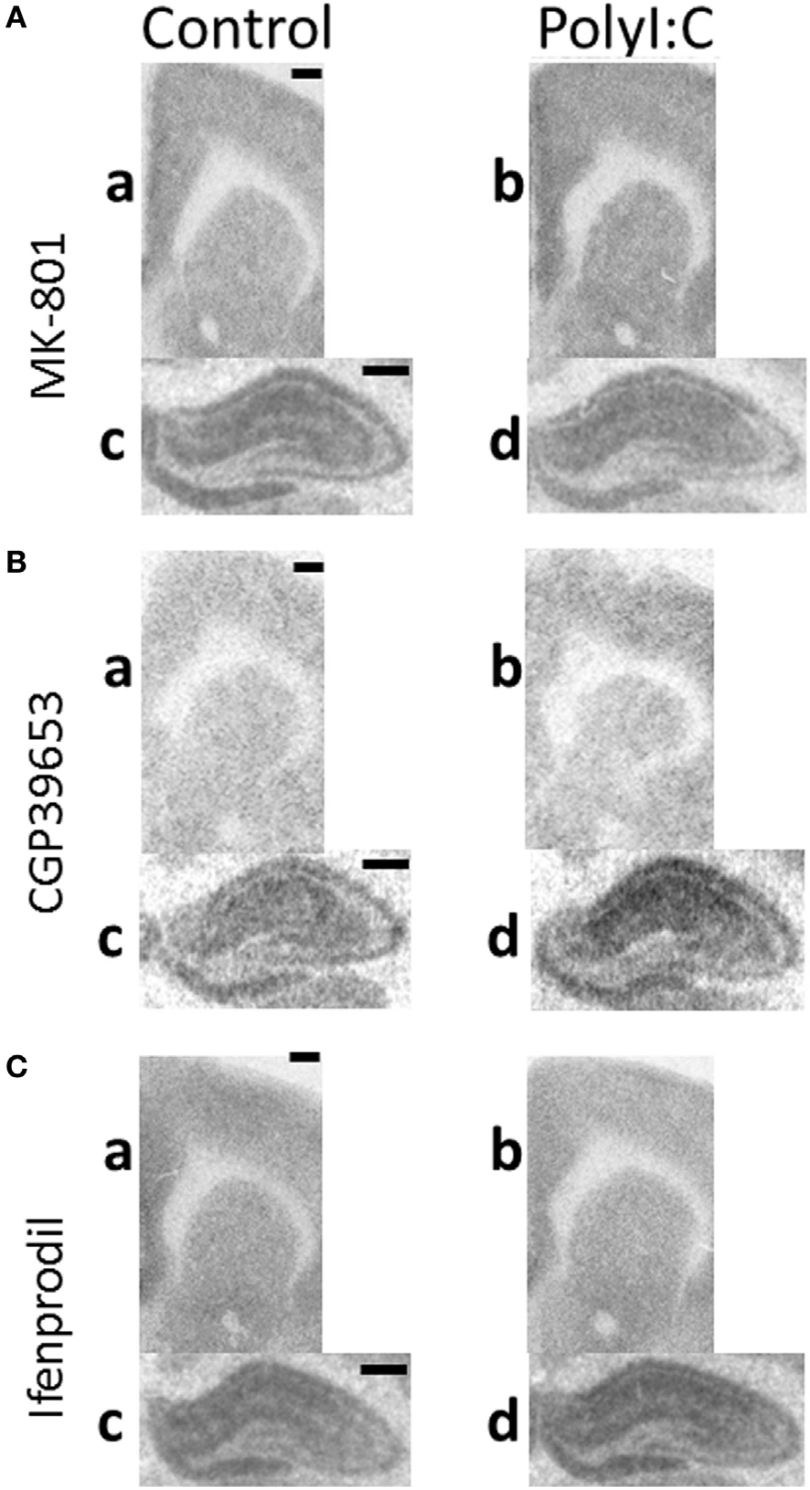
Representative autoradiographs of regions from maternal immune activation offspring. Coronal sections from adult (postnatal day 63–91) male offspring from vehicle (control: a,c) or polyI:C (b,d) treated dams were processed with tritiated radioligands to detect **(A)** *N*-methyl-d-aspartate receptor (NMDAR) channel (MK-801), **(B)** NR2A (CGP39653), and **(C)** NR2B (Ifenprodil) binding in (a,b: ~2.50 mm bregma) cortical, striatal, and (c,d: ~−3.10 mm bregma) hippocampal regions. **(A)** PolyI:C offspring showed significantly increased NMDAR channel binding in (a,b) cingulate and striatum, but not (c,d) hippocampal regions. **(B)** PolyI:C offspring showed significant increases in NR2A binding overall in all quantified regions. **(C)** PolyI:C offspring showed no significant change in NR2B binding overall in any quantified region. Scale bars represent 500 µm.

Representative autoradiographs of binding assays are shown in Figure 2 and show the expected pattern of NMDAR channel, NR2A subunit, and NR2B subunit (Figures 2A–C, respectively) binding and distribution in rodent brain (45–47). Briefly, signals are evenly distributed across the cortex and striatum. White matter tracts (i.e., corpus callosum) are not labeled and hippocampal architecture including neuropil areas surrounding ammon’s horns region are labeled. Overall, autoradiographic signals were less in cortex and striatum (a,b) in comparison to hippocampus (c,d).

#### [^3^H]MK-801 Binding (NMDAR Channel)

**Figure 3:**
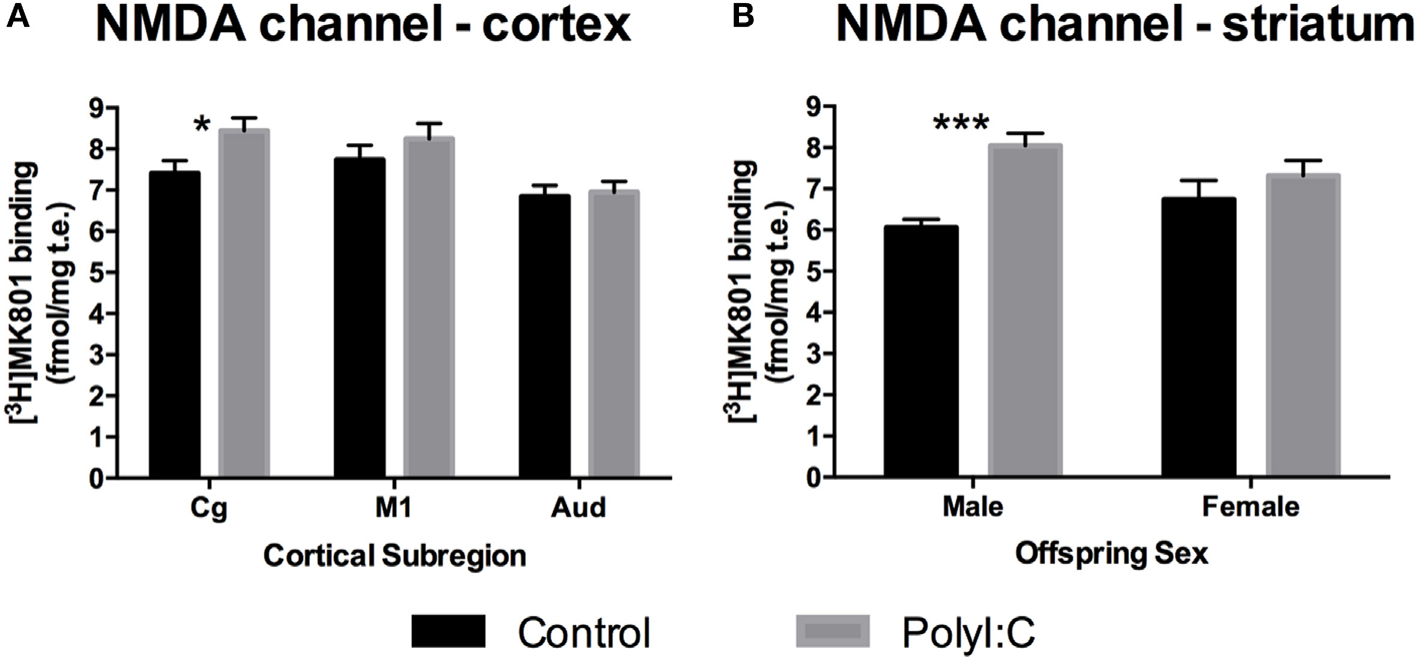
*N*-methyl-d-aspartate receptor (NMDAR) channel binding alterations in cortex and striatum from adult offspring exposed to vehicle (control; black) or polyI:C (gray) during early (GD10) or late (GD19) gestation. Tritiated MK-801 binding was used to quantify NMDAR channel levels. Significantly increased NMDAR binding was found in the cingulate subregion of polyI:C offspring compared to controls **(A)**. Male, but not female, polyI:C offspring specifically exhibited significantly increased NMDAR channel binding in the striatum **(B)**. Bars represent mean ± SEM (*n* = 15–28 rats per group). Cg, cingulate cortex; M1, motor cortex; Aud, auditory cortex (*p < 0.05, ***p < 0.001).

Although there was no overall effect of polyI:C on NMDAR channel binding ([^3^H]MK-801 binding) in the cortex [Treatment effect: *F*_(1,45)_ = 1.86, *p* > 0.10], polyI:C offspring had ~13% more NMDAR channel expression than control offspring in Cg [Treatment × Subregion effect: *F*_(2,90)_ = 3.27, *p* < 0.05; Control versus polyI:C in cingulate: *F*_(1,45)_ = 5.34, *p* < 0.05; Figures 2A and 3A]. PolyI:C did not affect NMDAR channel binding in the M1 or Aud (Figure 3A). PolyI:C did not affect NMDAR channel binding in the hippocampus [Treatment effect: *F*_(1,45)_ = 1.13, *p* > 0.10; Figure 2A; Table 3]. There was no effect of offspring sex or GD of exposure on NMDAR channel binding in either the cortex or the hippocampus [both, *F*_(1,42)_ < 2.65, *p* > 0.10].

**Table 3:** Overall effect of gestational polyI:C exposure on NMDA receptor channel and subunit binding.

Binding (fmol/mg tissue equivalent)		Mean ± SEM (*n*)		
		Control	Maternal immune activation	Treatment effect
*N*-methyl-d-aspartate receptor channel [^3^H]MK-801	Cortex	7.33 ± 0.29 (30)	7.87 ± 0.28 (23)	*F*_(1,45)_ = 1.86, *p* > 0.10
	Hippocampus	8.74 ± 0.3 (30)	9.15 ± 0.27 (23)	*F*_(1,45)_ = 1.13, *p* > 0.10
	Striatum	**6.5 ± 0.23 (29)**	7.62 ± 0.27 (25)***	*F*_(1,45)_ = 17.63, *p* < 0.001
NR2A [^3^H]CGP39653	Cortex	**2.69 ± 0.31 (28)**	4.24 ± 0.36 (25)**	*F*_(1,45)_ = 11.004, *p* < 0.01
	Hippocampus	**4.05 ± 0.28 (28)**	5.3 ± 0.42 (25)*	*F*_(1,44)_ = 6.22, *p* < 0.05
	Striatum	**1.69 ± 0.14 (26)**	2.3 ± 0.22 (25)*	*F*_(1,47)_ = 6.62, *p* < 0.05
NR2B [^3^H]Ifenprodil	Cortex	11.03 ± 0.36 (31)	10.77 ± 0.32 (27)	*F*_(1,50)_ = 0.24, *p* > 0.10
	Hippocampus	13.81 ± 0.38 (31)	13.58 ± 0.35 (25)	*F*_(1,48)_ = 0.11, *p* > 0.10
	Striatum	13.26 ± 0.6 (30)	13.14 ± 0.5 (24)	*F*_(1,47)_ = 0.08, *p* > 0.10

*p < 0.05

**p < 0.01

***p < 0.001

In the striatum, polyI:C offspring had ~20% more NMDAR channel binding than control offspring [Treatment effect: *F*_(1,45)_ = 17.63, *p* < 0.001; Table 3; Figure 2A]. In addition, male polyI:C offspring had ~34% more NMDAR channel binding than male control offspring [Treatment × Sex effect: *F*_(1,45)_ = 6.68, *p* < 0.05; Pairwise contrast: *F*_(1,45)_ = 22.68, *p* < 0.001; Figure 3B]. No change was observed in polyI:C-exposed compared to control females [*F*_(1,45)_ = 1.322, *p* > 0.05; Figure 3B]. There was no effect of GD of exposure on NMDAR channel binding in the striatum [*F*_(1,45)_ = 3.473, *p* < 0.10].

#### [^3^H]CGP39653 Binding (NR2A Subunit)

**Figure 4:**
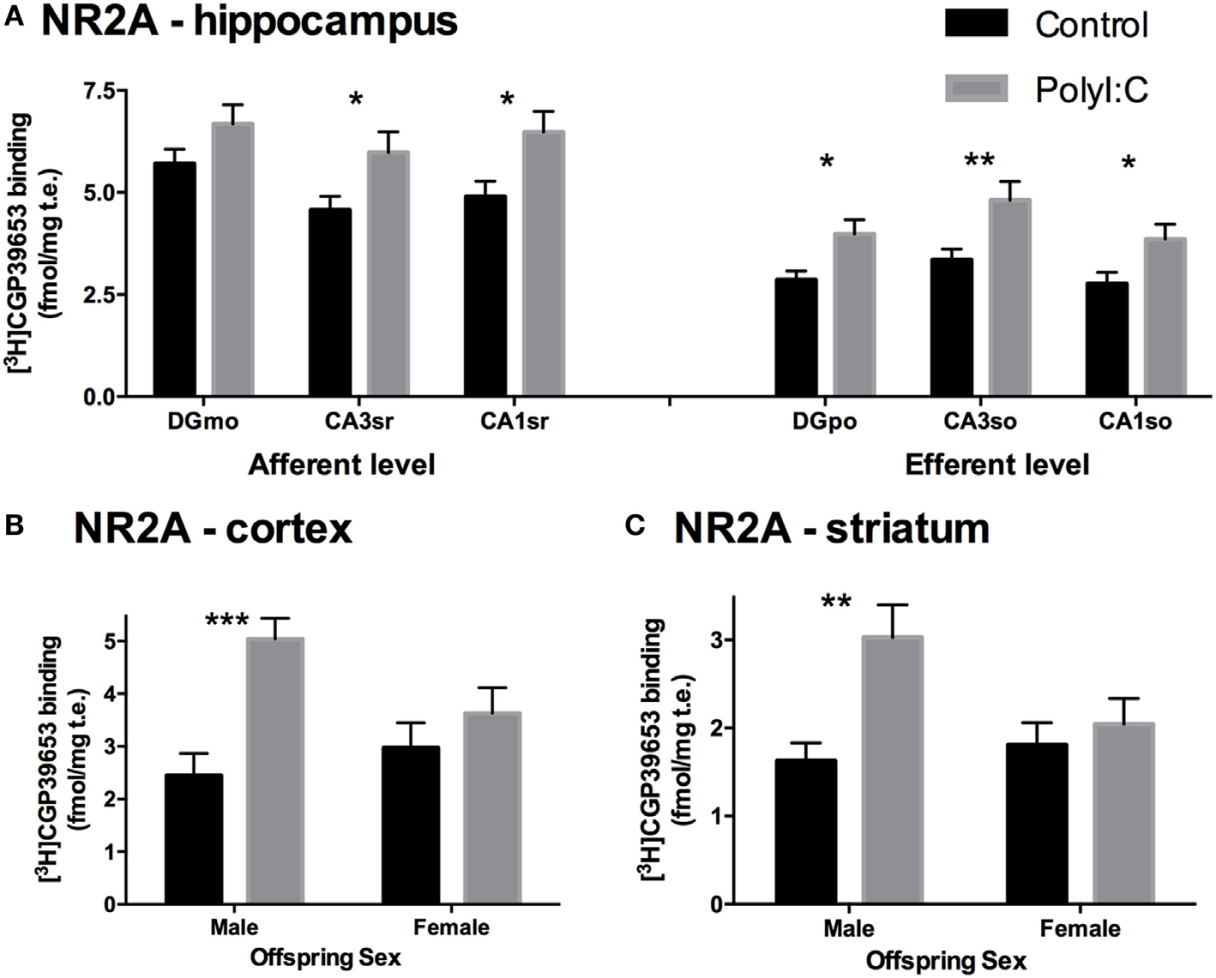
NR2A subunit binding alterations in hippocampus, cortex, and striatum from adult offspring exposed to vehicle (control; black) or polyI:C (gray) during early (GD10) or late (GD19) gestation. Tritiated CGP39653 was used to quantify NR2A subunit levels. PolyI:C offspring had significantly increased NR2A binding in all hippocampal subregional levels, except the DGmo **(A)**. Male, but not female, rats exposed to polyI:C during gestation had increased NR2A binding in the cortex **(B)** and striatum **(C)**. Bars represent mean ± SEM (*n* = 15–28 rats per group). DGmo, molecular layer of dentate gyrus; CA3sr, stratum radiatum of cornu ammonis 3; CA1sr, stratum radiatum of cornu ammonis 1; DGpo, polymorph layer of dentate gyrus; CA3so, stratum oriens of cornu ammonis 3; CA1so, stratum oriens of cornu ammonis 1 (*p < 0.05, **p < 0.01, ***p < 0.001).

In total hippocampus, polyI:C offspring had ~30% more NR2A binding than control offspring [Treatment effect: *F*_(1,44)_ = 6.22, *p* < 0.05; Table 3; Figure 2B], the magnitude of this increase was moderate to strong (31–44% increases) in all subregions [Treatment × Subregion × Level effect: *F*_(1.7,45)_ = 3.68, *p* < 0.05; Pairwise contrasts: *F*_(1,44)_ > 5.41, *p* < 0.05; Figure 4A] except the molecular layer of the DG [Pairwise contrast: *F*_(1,44)_ = 2.74, *p* > 0.10; Figure 4A]. There was no effect of gestation day of exposure on NR2A binding in the hippocampus, cortex, or striatum [*F*_(1,45–47)_ < 0.178, *p* > 0.10].

Overall, polyI:C offspring had ~57% more NR2A binding than control offspring in the cortex [Treatment effect: *F*_(1,45)_ = 11.004, *p* < 0.01; Table 3; Figure 2B]. In addition, we detected a sex by treatment interaction effect, where male polyI:C offspring had ~132% more NR2A binding than male control offspring in cortical tissue [Treatment × Sex effect: *F*_(1,45)_ = 4.794, *p* < 0.05; Pairwise contrast: *F*_(1,42)_ = 24.61, *p* < 0.001; Figure 4B], an effect that was not observed for females [*F*_(1,45)_ = 0.483, *p* > 0.10; Figure 4B].

Overall, polyI:C offspring had ~43% more NR2A binding than control offspring in the striatum [Treatment effect: *F*_(1,47)_ = 6.62, *p* < 0.05; Table 3; Figure 2B]. Similar to the cortex, polyI:C-exposed male offspring had ~86% more NR2A binding than male control offspring [Treatment × Sex effect: *F*_(1,47)_ = 5.38, *p* < 0.05; Pairwise comparison: *F*_(1,47)_ = 11.79, *p* < 0.01; Figure 4C], an effect which was not observed in females [*F*_(1,45)_ = 0.03, *p* > 0.10; Figure 4C].

#### [^3^H]Ifenprodil Binding (NR2B Subunit)

There was a consistent lack of change in [^3^H]Ifenprodil expression across all regions in polyI:C offspring compared to control offspring (Table 3; Figure 2C). There was no effect of treatment, or any interaction of treatment with offspring sex, GD of exposure, or anatomical region in the cortex [all *F*_(1,50)_ and *F*_(2,100)_ < 0.50, *p* > 0.10; Table 3; Figure 2C], hippocampus [all *F*_(1,48)_ < 0.40, *F*_(1.4,62.8)_ < 1.50, *F*_(2,96)_ < 2.21, *p* > 0.10; Table 3; Figure 2C], or striatum [all *F*_(1,47)_ and *F*_(2,94)_ < 0.50, *p* > 0.05; Table 3; Figure 2C] on NR2B binding.

#### NR2A:NR2B Ratio in Hippocampus

**Figure 5:**
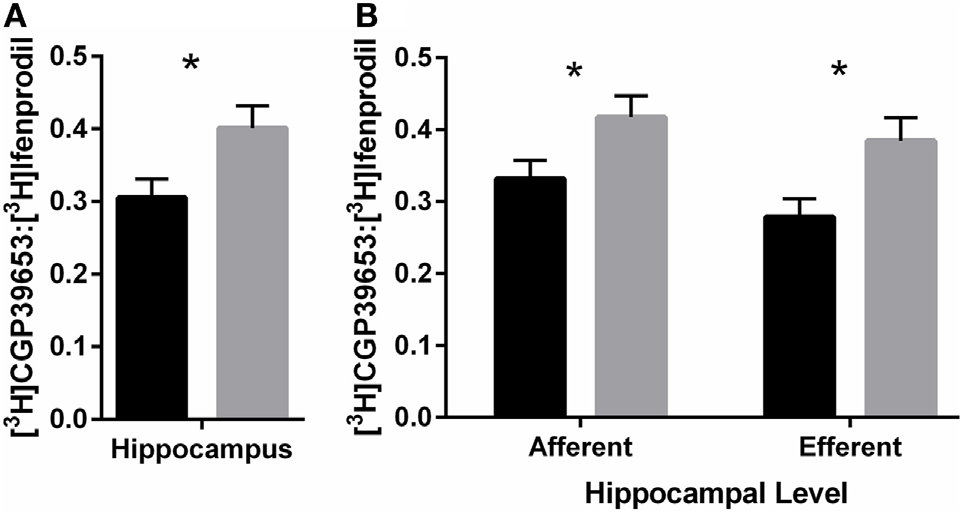
NR2A:NR2B binding ratio in hippocampus of adult offspring exposed to vehicle (control; black) or polyI:C (gray) during early (GD10) or late (GD19) gestation. Tritiated CGP39653 and Ifenprodil were used to calculate the ratio of NR2A:NR2B subunit binding. **(A)** The ratio of NR2A:NR2B ratio binding in the hippocampus was significantly increased in polyI:C offspring. **(B)** This increased ratio was elevated at both afferent and efferent levels of each hippocampal subregion. Bars represent mean ± SEM (*n* = 25–28 rats per group) (*p < 0.05).

PolyI:C offspring had ~31% increased NR2A:NR2B ratio compared to control offspring [Treatment effect: *F*_(1,46)_ = 5.40, *p* < 0.05; Figure 5A]. In addition, compared to control offspring, polyI:C offspring had ~25% higher NR2A:NR2B ratio in hippocampal afferent levels [*F*_(1,46)_ = 4.35, *p* < 0.05] and ~38% higher NR2A:NR2B ratio in hippocampal efferent levels [*F*_(1,46)_ = 6.37, *p* < 0.05] than control offspring [Treatment × Level effect: *F*_(1,46)_ = 5.09, *p* < 0.05; Figure 5B].

### Effect of Maternal PolyI:C Treatment on NR1 and NR2A mRNA in Adult Male Offspring

**Figure 6:**
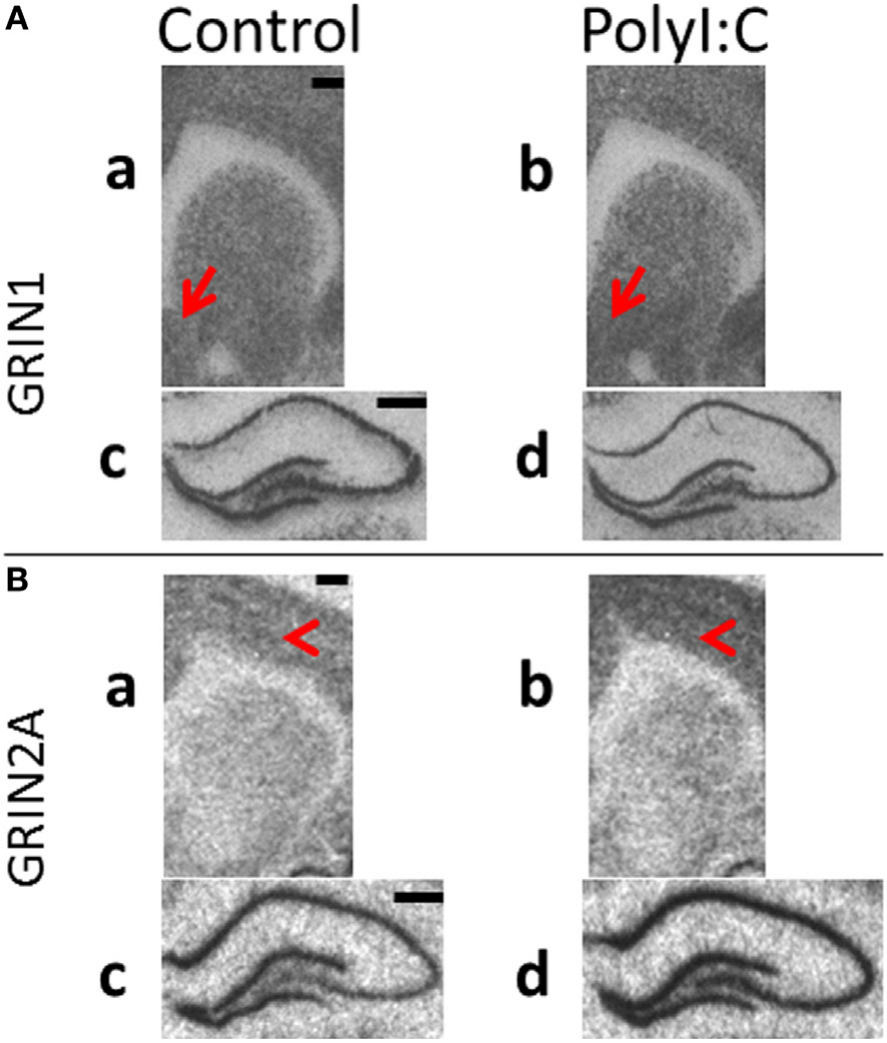
Representative *in situ* hybridization films of *N*-methyl-d-aspartate receptor subunit gene expression in brain regions from male polyI:C offspring. Coronal sections from adult (postnatal day 63–91) male offspring from vehicle (control: a,c) or polyI:C (b,d) treated dams were processed to detect **(A)** GRIN1 (NR1) and **(B)** GRIN2A (NR2A) mRNA in (a,b: ~2.30 mm bregma) cortical, striatal and (c,d: ~−3.10 mm bregma) hippocampal regions. **(A)** PolyI:C offspring showed significant increases in NR1 mRNA in (a,b) nucleus accumbens shell (red arrows), with no significant changes in cortex or (c,d) hippocampus. **(B)** PolyI:C offspring showed (a,b) no significant change in NR2A mRNA in striatum, but significantly elevated NR2A mRNA in cortex (red arrowheads) and (c,d) hippocampal regions. Scale bars represent 500 µm.

Representative *in situ* autoradiographs are shown in Figure 6 and the expected pattern of NR1 mRNA (GRIN1, Figure 6A) and NR2A mRNA (GRIN2A, Figure 6B) distribution was identified in rodent brain (37). Briefly, signals were evenly distributed across the cortex and striatum (a,b). In the hippocampus, the granular layer of the DG and the pyramidal neuronal layers within ammons-horn (CA3–CA1) were darkly labeled for both NMDAR1 and NMDAR2A mRNAs (c,d), whereas the hilar region of CA4 had intermediate levels of both mRNAs. NR2A mRNA (Figure 6B) was less in the striatum relative to the cortex (a,b). White matter tracts were clearly identifiable (corpus callosum and anterior commissure) with no mRNA signal. Overall, all autoradiographic signals were less intense and more homogenous in cortex and striatum (a,b) in comparison to the hippocampus (c,d).

**Figure 7:**
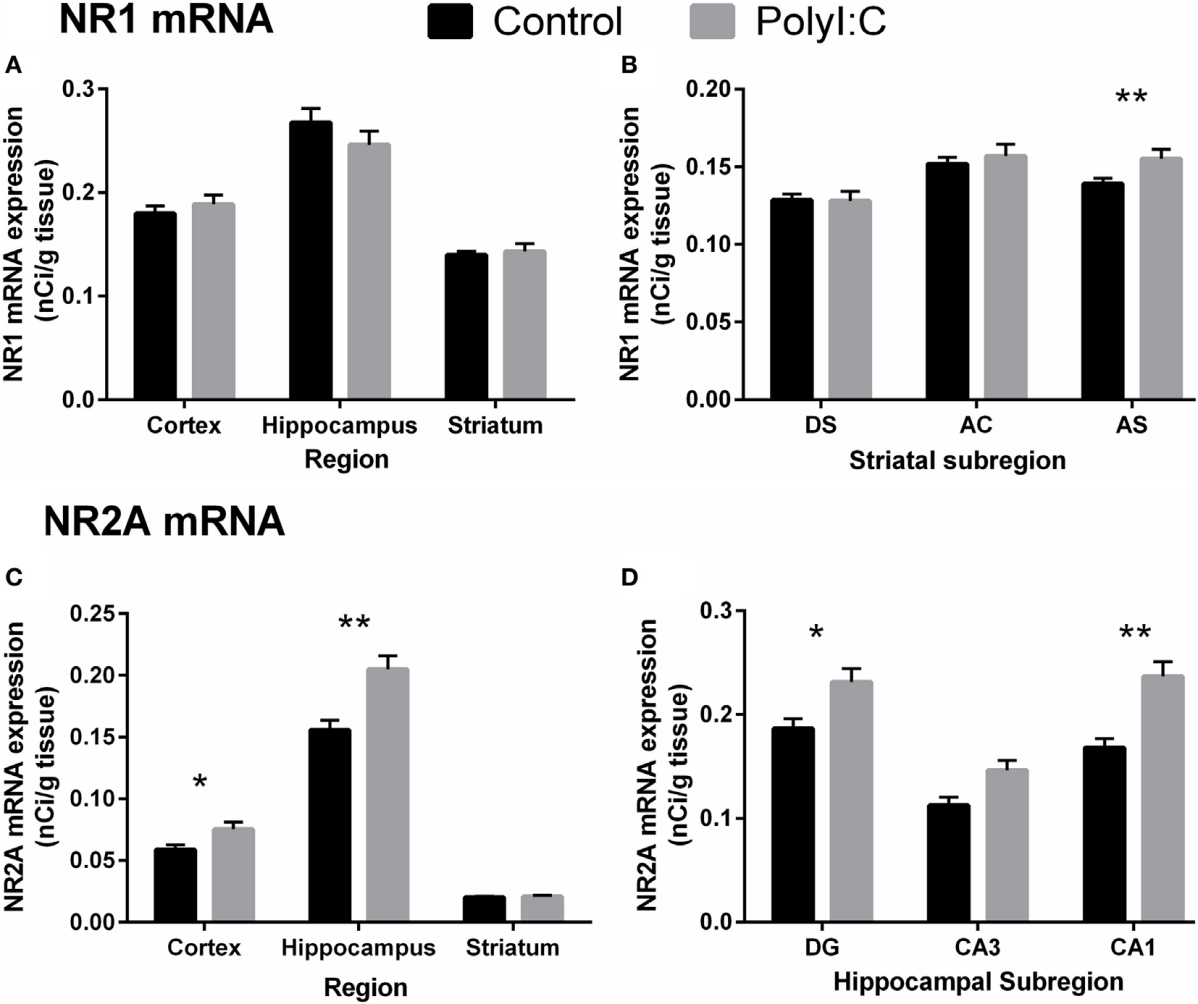
*N*-methyl-d-aspartate receptor-related mRNA alterations in cortex, hippocampus, and striatum from male adult offspring exposed to vehicle (control; black) or polyI:C (gray) during early (GD10) or late (GD19) gestation. *In situ* hybridization was used to quantify NR1 mRNA (GRIN1; **A,B**) and NR2A mRNA (GRIN2A; **C,D**). Male polyI:C offspring had **(A)** no change in NR1 mRNA overall in any quantified region, but **(C)** significantly increased NR2A mRNA in cortex and hippocampus. **(B)** Male polyI:C offspring had significantly elevated NR1 mRNA in, the striatal subregion, AS. **(D)** PolyI:C offspring had significantly increased NR2A mRNA, in the hippocampal subregions, DG, and CA1. Bars represent mean ± SEM (*n* = 25–28 rats per group). DG, dentate gyrus; CA3, cornu ammonis 3; CA1, cornu ammonis 1; DS, dorsal striatum; AC, nucleus accumbens core; AS, nucleus accumbens shell (*p < 0.05, **p < 0.01).

Maternal immune activation did not affect NR1 mRNA levels in cortical tissue [all Treatment effects/interactions: *F*_(1,22)_ < 2.88, *p* > 0.05; Figures 6A and 7A]. Similarly, no treatment main effects or interactions were observed for NR1 mRNA in hippocampus [all *F*_(1,24)_ < 2.64, all *F*_(1.4,24)_ < 1.0, *p* > 0.05; Figure 7A]. In the striatum, polyI:C offspring had a small, but statistically significant, increase (~11%) in NR1 mRNA in the AS compared to controls [Treatment × Subregion: *F*_(2,44)_ = 6.08, *p* < 0.01; Pairwise contrast: *F*_(1,22)_ = 8.11, *p* < 0.01; Figures 6B and 7B].

In contrast to the overall lack of large changes in NR1 mRNA, polyI:C offspring had widespread increases in NR2A mRNA. Compared to controls, polyI:C offspring had ~25% more NR2A mRNA in cortex [Treatment effect: *F*_(1,24)_ = 4.20, *p* < 0.05] and ~22% more NR2A mRNA in hippocampus [Treatment effect: *F*_(1,24)_ = 9.02, *p* < 0.01; Figures 6B and 7C]. In addition, polyI:C offspring had ~18% more NR2A mRNA in DG [*F*_(1,24)_ = 4.53, *p* < 0.05], and ~29% more NR2A mRNA in CA1 [*F*_(1,49)_ = 16.61, *p* < 0.01; Subregion × Treatment effect: *F*_(2,48)_ = 3.28, *p* < 0.05; Figures 6B and 7D]. The increase of NR2A mRNA in CA3 of polyI:C offspring did not reach significance [*F*_(1,24)_ = 3.32, *p* < 0.10] (Figure 7D). In striatal tissue, there was no significant treatment or between-groups interaction effect [*F*_(1,23)_ < 3.15, *p* > 0.05; Figures 6B and 7C].

## Discussion

We found NMDAR-related molecular alterations in multiple brain regions from polyI:C offspring that potentially could contribute to early and late MIA behavioral changes previously reported in this model. However, contrary to our expectations, we did not find any interaction effects between the impact of MIA and GD. Additionally, while we would have expected decreased NMDAR binding overall, we found *increased* NMDAR channel binding, specifically in the Cg of polyI:C offspring. We also found novel and widespread increases in NR2A binding supported by significant increases in NR2A mRNA, along with no changes in NR2B binding, in polyI:C offspring. As hypothesized, we found more exaggerated molecular alterations in male polyI:C offspring, compared to female polyI:C offspring. Thus, we found that MIA elicited coordinated NMDAR alterations that were primarily in male offspring, in line with the exaggerated behavioral change found in males after MIA in our previous investigation (30).

Our most robust finding was increased NR2A binding in the brain of adult male polyI:C offspring. NR2A-rich NMDARs have relatively shorter channel opening duration (38), and faster decay kinetics (37, 48). We did not detect corresponding altered NMDAR channel binding in the hippocampus of polyI:C offspring, suggesting that more NMDARs would be of the NR1–NR2A containing type but may not be dramatically changed in overall numbers. This concurs with our finding of increased NR2A mRNA, and no change in NR1 mRNA, in several brain regions. The lack of interaction between treatment and gestational timing indicates that hippocampal NMDARs are potentially vulnerable to MIA at either gestational stage and suggests that increases in NMDAR2A could be a common brain response to developmental activation of the immune system. Previous studies report increased NR2A mRNA in the hippocampus of adult rats (7–8 weeks of age) exposed postnatally (at 2 weeks of age) to polyI:C (49) or the bacterial mimic LPS (50), suggesting that this NMDAR2A response to immune activation can even occur if the inflammation occurred after birth. This demonstrates that the mechanisms that govern NR2A expression in adulthood are potentially vulnerable to long-lasting effects of inflammation, regardless of the precise timing of inflammatory stress. In contrast to our lack of change in NR1 mRNA in the hippocampus, previous studies have reported ~20% reduced NR1 mRNA in the hippocampus of polyI:C mouse offspring exposed to late MIA, but not early MIA (13). Our data in rats suggest that polyI:C treatment during gestation may not affect NR1 gene expression to the same extent or as consistently as it does NR2A gene expression in the hippocampus. These variable findings could be due to subtle differences in the timing of vulnerable neurodevelopmental epochs, or species differences.

Our results show that the CA1 subregion exhibited increased NR2A mRNA and increased NR2A binding and NR2A:NR2B ratio in the apical dendritic region (afferent) in polyI:C offspring, regardless of gestational timing of MIA and offspring sex. This suggests that synaptic input coming from CA3, which is critically dependant on NMDAR function for learning and memory (Schaffer collaterals), is significantly changed after developmental immune activation. Indeed, increases in NR2A:NR2B ratio reduces both long-term potentiation and long-term depression in the CA3-to-CA1 circuitry [see Ref. (38) for details], limiting plasticity and making it more difficult to learn and remember. Our present findings therefore suggest that polyI:C offspring have associated molecular alterations in CA1 that perhaps would produce longer term memory retrieval deficits, consistent with long-term memory deficits found in adult polyI:C rat offspring (26).

We find localized increases in NMDAR binding in the Cg of polyI:C offspring, suggesting that MIA can elicit long-lasting changes in NMDAR expression within cortical regions of adult offspring, regardless of sex or gestational timing of MIA. Although systemic MK-801 did not alter locomotion in polyI:C offspring in our previous studies (30), other studies have reported both increased (18) and decreased (19–21) locomotion in polyI:C offspring in response to systemic MK-801. The mechanism by which MK-801 elicits behavioral changes, and the contribution of other neurotransmitter systems, is unclear (51–54). Although the increased NMDARs in the cingulate may not be sufficient to elicit consistent MK-801 locomotion alterations in all cohorts, it may impact cognition dependent on Cg. Indeed, previous studies report increased glutamatergic processes (55) and NMDAR channel binding in anterior (42) and posterior (56) Cg of people with schizophrenia.

We found some interesting changes within the striatal brain areas examined. Our current investigation of exaggerated molecular changes in striatal NMDAR of males appears to align with our previous behavioral investigation that showed that male, but not female, polyI:C rat offspring exhibit sensorimotor gating deficits (30). Our current molecular data shows that male, but not female, polyI:C offspring had increased NMDAR channel binding in the striatum, and elevated NR2A binding in both the cortex and striatum. Given that these molecular and behavioral alterations were exaggerated in male polyI:C offspring, one interpretation would be that these cortical and striatal changes contribute to the sensorimotor gating deficits that male polyI:C offspring exhibit at adulthood. However, further research is needed to elucidate how MIA-induced changes in NR2A-rich NMDARs are modified by sex and how NMDARs may contribute to sensorimotor gating function. In the present study, the coordinated increase in striatal NR2A and NMDAR channel binding in male polyI:C offspring indicates they potentially have increased NR2A-rich NMDARs in the striatum. In normal rodents, the “indirect” striatopallidal pathway is primarily mediated by NR1–NR2A-rich NMDARs, while the direct “striato-nigral–entopeduncular” pathway is mediated by NR1–NR2B rich NMDARs (57–59). The changes in NMDARs in the MIA model could suggest more activity in the indirect striatopallidal pathway, which is also known to be enriched in D2 receptors. However, how the striatal changes found here map onto these distinct striatal pathways needs to be determined. PolyI:C offspring show increased dopamine turnover and reduced D2-like receptor levels in the striatum (60), supporting that the indirect striatal pathway may have more pathology in response to MIA, especially in male offspring. However, our previous investigation found no change in D2 receptor mRNA in the striatum of polyI:C offspring (30). This suggests that the NMDAR changes may precede or be independent of D2 receptor changes. Previously, we found increased D1 dopamine receptor mRNA in the nucleus accumbens of male GD10 polyI:C offspring (30). In this study, we found that male polyI:C offspring had increased NR1 mRNA in the nucleus accumbens shell only, regardless of gestational timing but with overall increases in striatal NMDAR channel binding. This suggests male polyI:C offspring may have elevated NMDAR synthesis and activity in the striatum, particularly in the ventral portion. Taken together, the results on striatal abnormalities suggest dopaminergic and glutamatergic alterations found in early and/or late MIA rat offspring may interact and perhaps be rescued by pharmacotherapies that target either dopamine [as evidenced in MIA rat offspring treated with antipsychotics (18, 27)] or NMDAR, particularly NMDAR2A.

The primary finding of this study is that immune challenge *in utero* irrespective of timing of the challenge has an effect on glutamatergic signaling in adult offspring, an effect that seems to be largely restricted, or more extreme, in male offspring. In terms of the validity of the MIA model of schizophrenia, the sex differences are consistent with epidemiological and clinical evidence of a small increased incidence of schizophrenia in males and a more chronic course of the disorder in males (61). The long-term changes in NMDAR, particularly the consistent, anatomically widespread and quite robust increase in the NMDAR2A subunit may have deleterious effects on synaptic plasticity, learning and memory. We suggest that even transient developmental immune activation mediates neural and possibly behavioral changes, through adult changes in the synthesis and function of NMDAR. The clear and robust increases in NMDAR2A, whose gene has been recently linked to the increased risk of schizophrenia (62), suggest a possible point of convergence between genetic and environmental risk (maternal infection) factors in schizophrenia. Our study that identifies increased NMDAR2A after MIA suggests that future efforts could focus on determining when this NMDAR2A change occurs developmentally and could test if and when reversing the NMDAR2A increase could restore normal behavior.

## Ethics Statement

Experiments were performed in accordance with the National Health and Medical Research Council’s *Australian code for the care and use of animals for scientific purposes*. The current study was approved by the University of Newcastle’s Animal Care and Ethics Committee (Approval number A-2009-108).

## Availability of Data and Material

The data analyzed during the current study are available from the corresponding author on reasonable request.

## Author Contributions

KZ, US, JT, DH, and PM conceived and designed the experiments. CW assisted in the design of the experiments. LH and CM undertook the PolyI:C treatments and provided the rat brain tissue. KZ undertook the binding experiments, GRIN1 in situ experiment, contributed to the analysis of the hippocampus data, and provided a first draft based on hippocampus data only. TR undertook the GRIN2A experiment and analyzed the data. TR, TP-T, and CW wrote the final draft. TP-T and LH checked the data and edited the manuscript. All authors contributed to the interpretation and approval of the final manuscript.

## Conflict of Interest Statement

CW is a member of an Advisory Board for Lundbeck Australia Pty Ltd. The other authors declare that they have no competing interests.

## Footnotes

^1^http://rsb.info.nih.gov/nih-image.

^2^https://imagej.nih.gov/ij/.

## Abbreviations

Aud, auditory cortex; Cg, cingulate cortex; DS, dorsal striatum; M1, motor cortex; MIA, maternal immune activation; AC, nucleus accumbens core; AS, nucleus accumbens shell; DG, dentage gyrus; CA1, cornu ammonis area 1; CA3, cornu ammonis area 3; so, stratum oriens; sr, stratum radiatum; PBS, phosphate-buffered saline; NMDAR, *N*-methyl-d-aspartate receptor; polyI:C, polyriboinosinic:polyribocytidilic acid; GD, gestational day; PND, postnatal day; RM-ANOVA, repeated measures two-way analysis of variance.
